# Prediction of refractive error in adolescents using a multimodal large language model

**DOI:** 10.3389/fmed.2026.1770007

**Published:** 2026-03-10

**Authors:** Chaojun Chen, Yaqi Wang, Xia Zhang, Jiahong Han, Pinghui Hu, Jianjun Liu, Leilei Cao

**Affiliations:** 1 Department of Ophthalmology and Optometry, Lixiang Eye Hospital of Soochow University, Suzhou, Jiangsu, China; 2 Innovation Center of Yangtze River Delta, Zhejiang University, Jiaxing, Zhejiang, China

**Keywords:** artificial intelligence, fundus imaging, multimodal large language model, myopia prevention and control, pediatric refractive error

## Abstract

**Background:**

This study aims to develop and evaluate a multimodal large language model (LLM) for predicting refractive error (diopter) in adolescents by integrating fundus images with clinical and demographic data. The goal is to demonstrate how such a model can serve as a digital health tool for early screening and personalized management of refractive conditions in clinical and remote health settings.

**Methods:**

A dataset of 16,226 annotated records from adolescents aged 2 to 18 years was used. A vision-language foundation model based on Qwen2.5-VL was fine-tuned using supervised learning, incorporating both imaging and clinical data to predict spherical equivalent (SE). The model was trained on a randomly split dataset, and performance was evaluated using mean absolute error (MAE), root mean square error (RMSE), coefficient of determination (*R*^2^) and the Pearson correlation coefficient.

**Results:**

The model achieved a mean absolute error of 0.647 diopters and showed strong predictive performance across most refractive subgroups. The incorporation of multimodal data significantly outperformed single-modality models. Visualization analyses confirmed that the model's predictions are clinically interpretable and reliable.

**Conclusions:**

This multimodal LLM demonstrates the potential of digital health technologies to enhance refractive error prediction in adolescents. Its integration into digital health platforms can provide a non-invasive, scalable solution for early myopia screening, making it an invaluable tool for clinicians in both remote and urban settings.

## 1 Introduction

Refractive errors, including myopia, hyperopia, and astigmatism, are among the most common visual disorders in adolescents (1), affecting millions worldwide. Myopia, in particular, has seen a dramatic increase in prevalence over the past few decades, becoming a major global public health concern (2). This trend is especially pronounced in East Asia, where childhood myopia rates are strikingly high. The development of refractive errors in young populations is influenced by multiple factors, including genetics, education, lifestyle, and environmental conditions. The increasing prevalence of refractive errors in children and adolescents poses long-term consequences for visual function, educational performance, and ocular health, representing a growing public health challenge worldwide (3). Preventing the onset of myopia, slowing its progression, and reducing the risk of pathological myopia and associated complications have therefore become urgent global health priorities. Early detection of refractive abnormalities and accurate prediction of myopia risk are critical steps in effective prevention and control (4, 5).

Currently, vision screening in children and adolescents is primarily conducted through routine school-based eye examinations. While valuable, these programs face several limitations: high costs of personnel and equipment, variability in accuracy, delayed feedback, and relatively high misdiagnosis rates. Abnormal screening results often necessitate further examinations, such as cycloplegic refraction, which are time-consuming, inconvenient, and heavily dependent on the expertise of trained optometrists and access to specialized devices (6, 7). In underdeveloped regions, limited healthcare resources and weaker awareness of eye health present additional barriers to early detection and timely intervention. These challenges highlight the urgent need for more efficient, accessible, and scalable solutions to refractive error detection in adolescents (8, 9).

Artificial intelligence (AI) offers great potential in this domain by enabling multidimensional and standardized analysis of ophthalmic and demographic data (10–12). AI-powered refractive error detection systems can reduce reliance on subjective refraction, lower costs, improve efficiency, and enhance accuracy. Moreover, by analyzing longitudinal data from school vision screenings, AI can provide predictive insights into the progression of myopia, thereby supporting clinicians in tailoring preventive and therapeutic strategies for adolescents. Although widespread deployment of AI-based refractive error detection requires validation through larger datasets and long-term follow-up studies, its clinical advantages have already attracted significant attention, positioning AI as a promising tool for scalable screening and precision eye care.

In recent years, machine learning and deep learning have shown great promise in ophthalmology, with models leveraging diverse inputs such as optical coherence tomography (OCT), corneal topography, and axial length (13–15). However, most prior studies rely on a single modality, which limits generalizability in complex clinical scenarios (16, 17). Advances in multimodal learning–integrating multiple data types such as imaging and biometric measurements–have demonstrated improved performance across various areas of healthcare (18–20). Multimodal large language models (LLMs) (21, 22), in particular, represent a state-of-the-art approach capable of jointly encoding visual and structured data within a unified predictive framework (23–25). Despite successes in other ophthalmic applications (10, 14, 26), their use for refractive error prediction, especially in adolescents, remains underexplored .

The primary aim of this study is to develop and evaluate a multimodal LLM for predicting refractive error in adolescents by integrating fundus images (17) with key biometric and demographic data (27), including axial length, corneal curvature, age, and gender (28). We hypothesize that multimodal integration will improve prediction accuracy compared to traditional and single-modality approaches (29, 30). By leveraging a dataset of over 16,000 clinical records, this study seeks to provide evidence for the feasibility of AI-driven, scalable, and non-invasive refractive error prediction in pediatric populations. Ultimately, such approaches may help bridge the gap between school-based vision screening and clinical ophthalmology (31), offering an effective tool for early detection and public health interventions in myopia control.

## 2 Materials and methods

### 2.1 Data collection and statistical analysis

This study involving human participants was conducted in accordance with the ethical standards and with the Declaration of Helsinki. Ethical approval was obtained from the ethics committee of Lixiang Eye Hospital of Soochow University (reference SLER2025104). Informed consent was obtained from all individual participants included in the study. For participants who were under the age of 16, informed consent was explicitly obtained from their parents or legal guardians.

The dataset comprises 16,226 clinical records collected from 8,113 unique adolescent patients, aged 2 to 18 years, between August 2023 and January 2025. Each record contains key ocular and demographic information, including fundus images, patient age, gender, axial length, and corneal curvature. In addition, the spherical equivalent (SE) refractive error, determined through cycloplegic refraction using a combination of compound tropicamide and cyclopentolate hydrochloride, was measured by certified ophthalmologists or optometrists following standardized clinical procedures. This cycloplegic approach was adopted to temporarily paralyze accommodation, thereby eliminating the influence of accommodative spasm and ensuring the accuracy and reliability of refractive error measurements. The SE values thus obtained were included as the ground truth labels for model training and evaluation. Fundus images were acquired using a standardized retinal imaging device to ensure high resolution and consistency across all samples. Axial length and corneal curvature were measured using optical biometry devices such as the IOLMaster, while age and gender data were obtained through medical records or patient interviews.

For the purpose of statistical analysis, descriptive statistics were applied to summarize the distributions of key demographic variables (age and gender) and ocular biometric parameters (axial length, corneal curvature, and spherical equivalent). As shown in Table 1, the mean age of the participants was 9.0 years (Standard Deviation (SD) = 2.7), with a balanced gender distribution (4,180 males and 3,933 females). The mean axial length was 23.9 mm, and the mean corneal curvature was 43.3 diopters, consistent with expected norms for the adolescent population. The spherical equivalent (SE) refractive error, which served as the ground truth for model prediction, exhibited a mean of -0.96 D (SD = 2.65), indicating a myopia-dominant cohort. The distribution of SE values was left-skewed, reflecting a high prevalence of myopia among adolescents, especially in older age groups.

**Table 1:** Descriptive statistics of demographic and ocular parameters.

**Variable**	**Mean ±SD**	**Median (IQR)**	**Min**	**Max**
Age (years)	9.0 ± 2.7	9.0 (7.0, 11.0)	2.0	18.0
Axial length (mm)	23.9 ± 1.3	23.9 (23.1, 24.7)	18.7	30.6
Corneal curvature (D)	43.3 ± 1.4	43.3 (42.4, 44.3)	25.5	48.7
Spherical equivalent (D)	-0.96 ± 2.65	-0.75 (-2.00, 0.50)	-16.50	15.50
Gender (male/female)	4,180/3,933			

**Figure 1:**
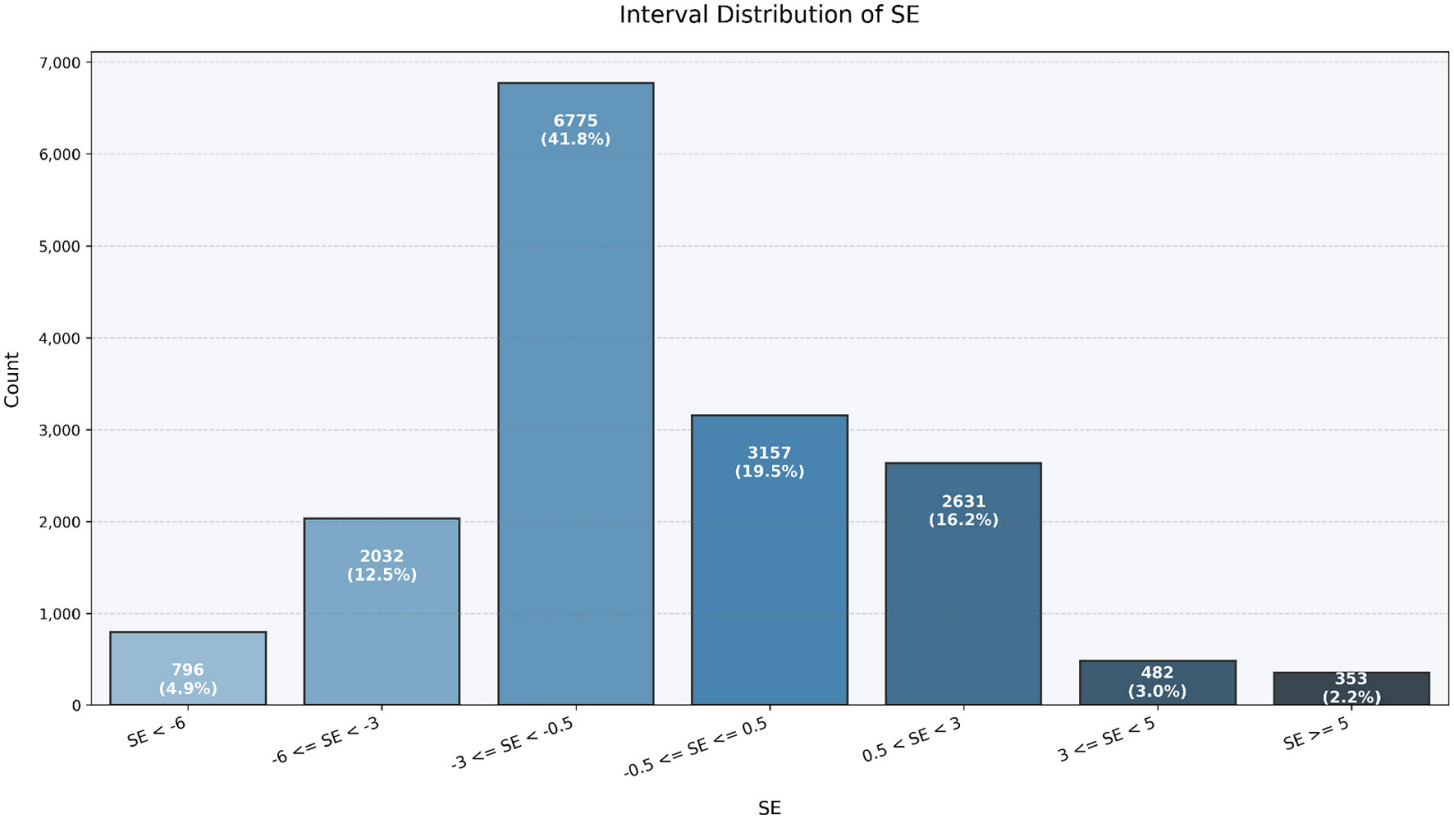
Interval-based distribution of spherical equivalent (SE) values in the study population. The majority of eyes are mildly to moderately myopic (−3.0*D* ≤ *SE* < −0.5*D*), with smaller proportions of near-emmetropic (−0.5*D* ≤ *SE* ≤ +0.5*D*) and hyperopic (*SE*>+0.5*D*) eyes. This distribution highlights the predominantly myopic nature of the cohort.

To further illustrate the distributional characteristics of refractive error in the study population, we visualized the interval-based frequency of the spherical equivalent (SE) as well as the bilateral SE comparison between the right eye (Oculus Dexter, OD) and left eye (Oculus Sinister, OS). As shown in Figure 1, the majority of eyes fell within the range of −3.0*D* to +0.5*D*. Notably, the interval −3.0*D* ≤ *SE* < −0.5*D* accounted for the largest proportion, comprising 41.8% of eyes, indicating a high prevalence of mild to moderate myopia. In addition, 19.5% of eyes fell within the range −0.5*D* ≤ *SE* ≤ +0.5*D*, representing near-emmetropic status. Moderate myopia (−6.0*D* ≤ *SE* < −3.0*D*) was observed in 12.5% of eyes, while 4.9% of eyes exhibited high myopia (*SE* < −6.0*D*). Hyperopia was relatively uncommon in this cohort, with 16.2% of eyes falling into the +0.5*Dto*+3.0*D* range, and only 3.0% and 2.2% of eyes in the +3.0*Dto*+5.0*D* and ≥+5.0*D* intervals, respectively. Overall, this distribution confirms that the study population is predominantly myopic, with a smaller proportion of hyperopic or near-emmetropic eyes.

**Figure 2:**
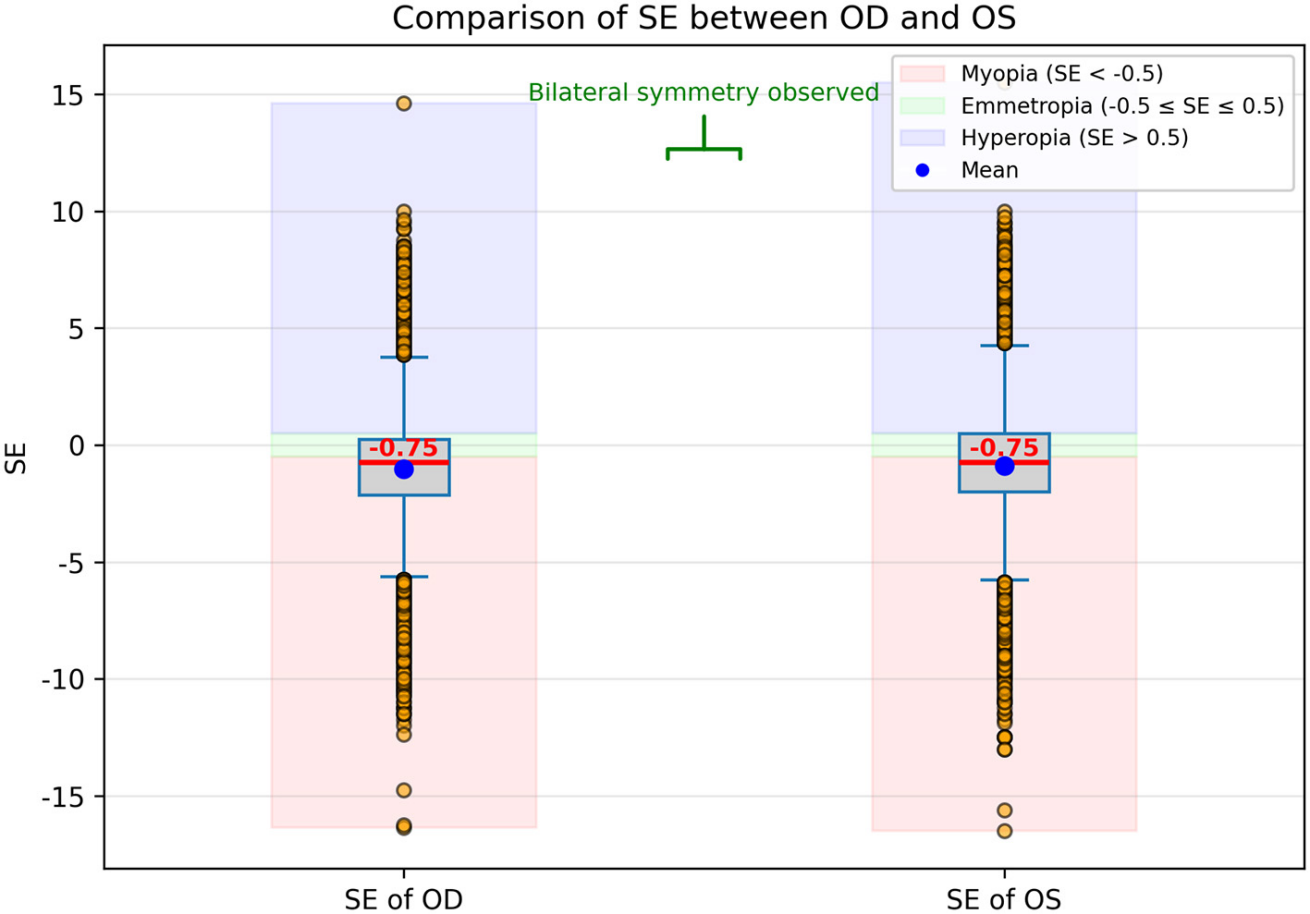
Boxplot comparison of spherical equivalent (SE) values between the right eye (OD) and left eye (OS). Each box represents the interquartile range (IQR, 25th-75th percentile), the horizontal line inside the box indicates the median SE, and the whiskers show the range of non-outlier values. Outliers are indicated as individual points beyond 1.5 × *IQR*. The distributions are largely symmetrical, with nearly identical median SE values between OD and OS, and comparable ranges and spreads, indicating high bilateral consistency in refractive status. Both OD and OS data were included in the model to maximize available information.

In addition, the comparison of SE values between OD and OS is presented in Figure 2 using boxplots. The distributions appear largely symmetrical, and the median SE values are nearly identical between the two eyes. The range, interquartile spread, and presence of outliers are also comparable, suggesting a high degree of bilateral consistency in refractive status, consistent with known physiological symmetry. Despite this interocular similarity, both OD and OS data were included in the model to maximize the use of available information. To avoid overfitting and ensure statistical independence during training and evaluation, we treated each eye as an independent sample and ensured that data from the same subject did not simultaneously appear in both training and validation/test sets. This approach allowed the model to benefit from the full dataset while minimizing the risk of data leakage.

### 2.2 Model description: Qwen2.5-VL

**Figure 3:**
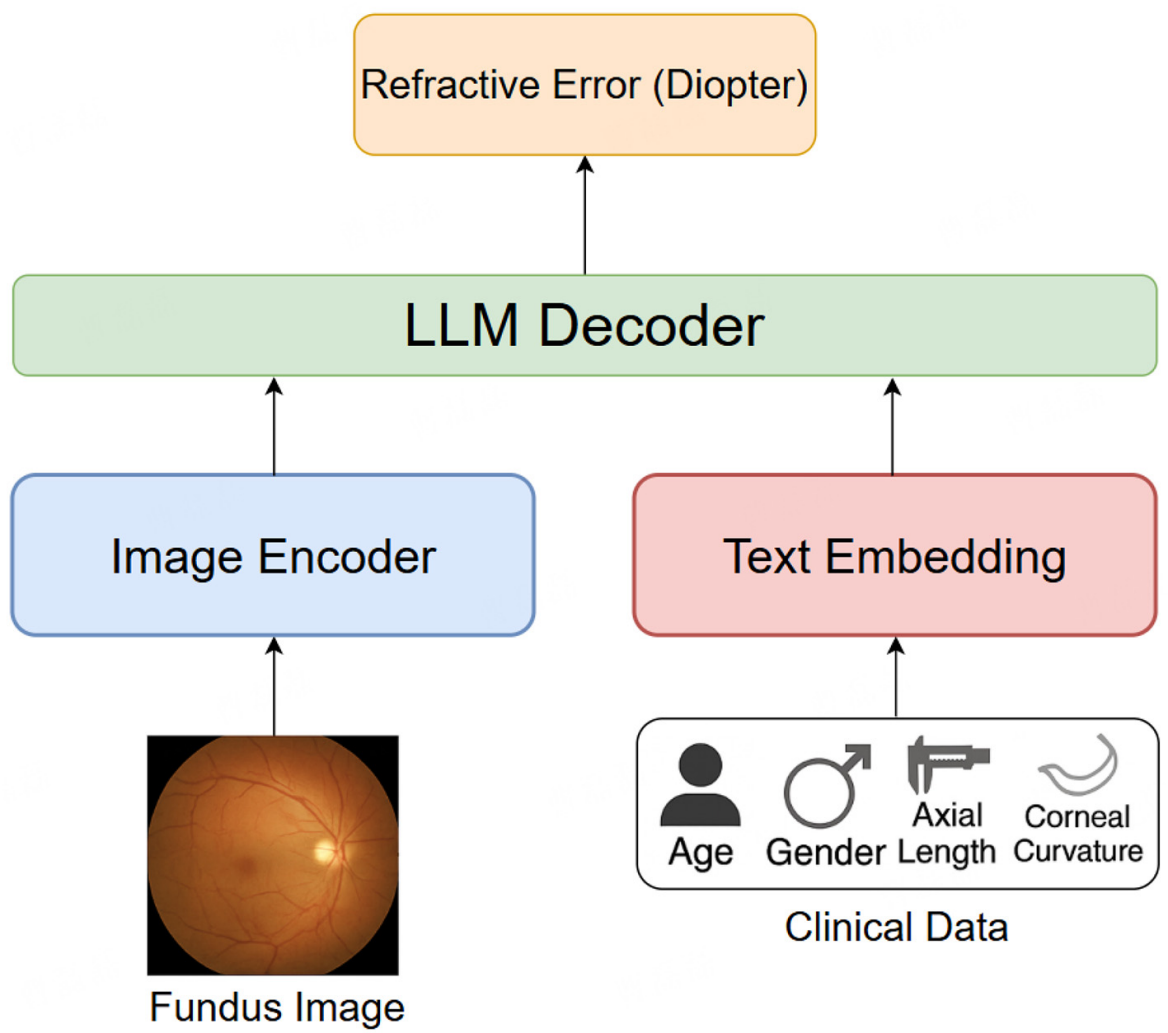
The whole pipeline of our proposed model for refractive error prediction.

The model used for refractive error prediction in this study is based on Qwen2.5-VL (32), a multimodal large language model (LLM) capable of jointly processing visual and non-visual clinical data. Qwen2.5-VL employs a unified architecture that integrates a Vision Transformer (ViT) for image encoding and a transformer-based language model for handling structured textual and numerical inputs. The whole pipeline of the proposed model is illustrated as Figure 3.

The ViT-based vision encoder is responsible for extracting high-level semantic features from fundus images. This approach divides each input image into patches and processes them via multi-head self-attention layers, capturing complex spatial and structural information from the retina that may correlate with refractive status. Meanwhile, the language transformer component processes patient metadata, including age, gender, axial length, and corneal curvature. These inputs are embedded and aligned with the visual representations through a cross-modal fusion mechanism, enabling the model to perform context-aware regression based on both anatomical imagery and biometric profiles.

This multimodal framework allows Qwen2.5-VL to effectively learn relationships between fundus morphology and clinical measurements, and to make robust predictions of spherical equivalent refractive error across a diverse pediatric population. The architecture of Qwen2.5-VL is designed to handle heterogeneous data types in a seamless manner, making it suitable for tasks like multimodal prediction in ophthalmology. This model employs attention mechanisms to integrate information from different modalities (images and structured clinical data), enabling the model to focus on the most relevant features from both the retinal images and the clinical parameters. Moreover, Qwen2.5-VL is capable of fine-tuning on specific tasks, allowing for highly specialized predictions like refractive error estimation based on individual patient data.

### 2.3 Image preprocessing and augmentation

All fundus photographs were processed using a standardized preprocessing pipeline prior to model training. Original images were captured at high resolution and resized to 512 × 512 pixels to match the input requirements of the vision encoder, while preserving the original aspect ratio through center cropping. Quality control was performed to exclude images with severe motion blur, defocus, occlusion, or illumination artifacts that could compromise retinal structure visibility. Images with incomplete retinal fields or unreadable metadata were also removed prior to analysis. No data augmentation was applied during model training. No histogram equalization or color normalization was applied. All preprocessed images were provided to the vision encoder of the Qwen2.5-VL model using the default image embedding configuration (Pixel intensity values were normalized to the range [0, 1] by dividing by 255. No histogram equalization or color normalization was applied.), without additional task-specific visual feature engineering.

### 2.4 Model training

To train the Qwen2.5-VL model effectively while balancing computational efficiency and model generalization, the dataset was randomly divided into two main subsets: 80% of the data was allocated for training, and the remaining 20% was reserved as an independent test set for final performance evaluation. Within the 80% training subset, 10% of the samples were further held out as a validation set, which was used to tune hyperparameters, monitor training convergence, and implement early stopping strategies. This stratified split ensured that both training and validation sets maintained similar distributions of demographic and biometric variables. The reserved test set, kept entirely unseen during training and validation, served as a reliable benchmark for assessing the model's generalization ability to new, previously unobserved data.

To fine-tune the multimodal model, we adopted Qwen2.5-VL-3B, a 3-billion-parameter variant of the Qwen2.5-VL family. Rather than full fine-tuning, we employed Low-Rank Adaptation (LoRA) to achieve parameter-efficient learning. The LoRA rank was set to 16, providing a balance between expressive capacity and efficiency. Model training was performed using the AdamW optimizer with an initial learning rate of 3e-4, combined with a cosine decay learning rate schedule and a linear warm-up during the first 10% of training steps. We trained the model with a batch size of 32 for a maximum of 12 epochs, using early stopping with a patience of 3 epochs based on validation MAE. Gradient clipping was set to 1.0, and mixed-precision training (FP16) was used to accelerate computation. All experiments were conducted using PyTorch, in combination with the Hugging Face Transformers and PEFT libraries, on a single NVIDIA RTX 4090 GPU. To ensure reproducibility, random seeds were fixed and data splits were consistent across training runs.

### 2.5 Evaluation metrics

The performance of the Qwen2.5-VL model was evaluated using several key metrics commonly used in regression tasks: mean absolute error (MAE), root mean squared error (RMSE), and coefficient of determination (*R*^2^). These metrics were selected to provide a comprehensive assessment of the model's predictive accuracy and reliability.

Mean Absolute Error (MAE): The primary evaluation metric was the MAE, which measures the average magnitude of the errors in the predicted refractive error without considering their direction. It is calculated as the average of the absolute differences between predicted and actual values. MAE is a robust metric that provides an intuitive understanding of the model's average prediction error in diopters.


$$\begin{array}{c} MAE=\frac{1}{n}\overset{n}{\underset{i=1}{\sum }}|y_{i}-{ŷ}_{i}| \end{array}$$ (1)

where *y*_*i*_ is the actual refractive error and ŷ_*i*_ is the predicted refractive error.

Root Mean Squared Error (RMSE): RMSE was used to assess the magnitude of error while giving higher weight to larger deviations. It is calculated by taking the square root of the average squared differences between predicted and actual values. RMSE provides an understanding of the model's performance in situations where larger errors are particularly undesirable.


$$\begin{array}{c} RMSE=\sqrt{\frac{1}{n}\overset{n}{\underset{i=1}{\sum }}{\left(y_{i}-{ŷ}_{i}\right)}^{2}} \end{array}$$ (2)

Unlike MAE, RMSE is more sensitive to outliers and large errors, making it useful for assessing the model's robustness in handling extreme cases.

Coefficient of Determination (*R*^2^): *R*^2^ was used to quantify how well the model's predictions fit the actual refractive error values. It measures the proportion of variance in the refractive error that is explained by the model. An *R*^2^ value closer to 1 indicates that the model is able to explain most of the variance in the data, while a value closer to 0 indicates poor predictive power.


$$\begin{array}{c} R^{2}=1-\frac{\sum_{i=1}^{n}{\left(y_{i}-{ŷ}_{i}\right)}^{2}}{\sum_{i=1}^{n}{\left(y_{i}-ȳ\right)}^{2}} \end{array}$$ (3)

where ȳ is the mean of the actual refractive errors.

These metrics were calculated on the test set to evaluate the final performance of the trained model.

In addition to error-based metrics, the Pearson correlation coefficient (r) was used to assess the linear association between predicted and ground-truth refractive errors. Pearson r reflects the consistency of prediction trends and is independent of the absolute scale of prediction errors. This metric is particularly informative for subgroup analyses with narrow spherical equivalent ranges, where the variance of ground-truth values is limited and the coefficient of determination (*R*^2^) may become less reliable or even negative. Therefore, Pearson r was reported as a complementary goodness-of-fit measure to better characterize model performance across refractive subgroups.

## 3 Results

### 3.1 Performance across refractive error groups

Table 2 presents the model's prediction performance across different refractive error (SE) groups in the test set, evaluated by Mean Absolute Error (MAE), Root Mean Square Error (RMSE), the coefficient of determination (*R*^2^) and Pearson r. The stratification includes seven SE-based categories: high myopia, moderate myopia, low myopia, emmetropia, low hyperopia, moderate hyperopia, and high hyperopia. Additionally, overall performance across all test samples is reported.

**Table 2:** Model performance across stratified refractive error groups in the test set, evaluated using MAE, RMSE, *R*^2^ and Pearson r.

**Group**	**Numbers**	**MAE (D)**	**RMSE (D)**	***R* ^2^**	**Pearson r**
High myopia	143	0.861	1.273	0.330	0.709
Moderate myopia	401	0.902	1.133	-1.056	0.652
Low myopia	1251	0.596	0.782	-0.464	0.536
Emmetropia	797	0.533	0.770	-4.153	0.397
Low hyperopia	478	0.580	0.763	-0.964	0.555
Moderate hyperopia	99	0.827	1.178	-3.534	0.338
High hyperopia	77	1.096	1.593	-1.283	0.631
All samples	3246	0.647	0.895	0.883	0.940

Grouping was based on spherical equivalent (SE). All error metrics are reported in diopters (D). Negative *R*^2^ values in certain subgroups reflect the limited variance and small sample sizes within those strata and should be interpreted with caution. Pearson correlation coefficients are reported to reflect prediction trend consistency within narrow refractive ranges, where *R*^2^ may be less informative.

The model achieved the lowest MAE (0.5331 D) in the emmetropic group and the highest MAE (1.0962 D) in the high hyperopia group. Similarly, RMSE ranged from 0.7627 D (low hyperopia) to 1.5931 D (high hyperopia). Interestingly, while the overall *R*^2^ value was 0.8833, indicating strong predictive power across the full dataset. In subgroup analyses, negative *R*^2^ values were observed in several refractive categories, particularly those with narrow SE distributions (e.g., emmetropia) or limited sample sizes (e.g., high hyperopia). This phenomenon is well recognized in regression analysis, as the coefficient of determination becomes unstable when the variance of ground truth values within a subgroup is low. In such cases, even modest absolute prediction errors may exceed the intrinsic variability of the target variable, resulting in negative *R*^2^ values. Therefore, absolute error metrics such as MAE and RMSE provide a more reliable and clinically meaningful evaluation of model performance at the subgroup level.

To further assess the linear relationship between predictions and ground truth, Pearson correlation coefficients (r) were calculated. Overall, the model achieved a strong correlation across all samples (r = 0.940). In subgroup analyses, Pearson r values ranged from 0.338 (moderate hyperopia) to 0.709 (high myopia), reflecting varying linear consistency in different SE groups. Notably, the trends in Pearson r were generally consistent with MAE and RMSE, confirming that the model maintains reasonable predictive reliability even in subgroups where *R*^2^ is unstable.

**Figure 4:**
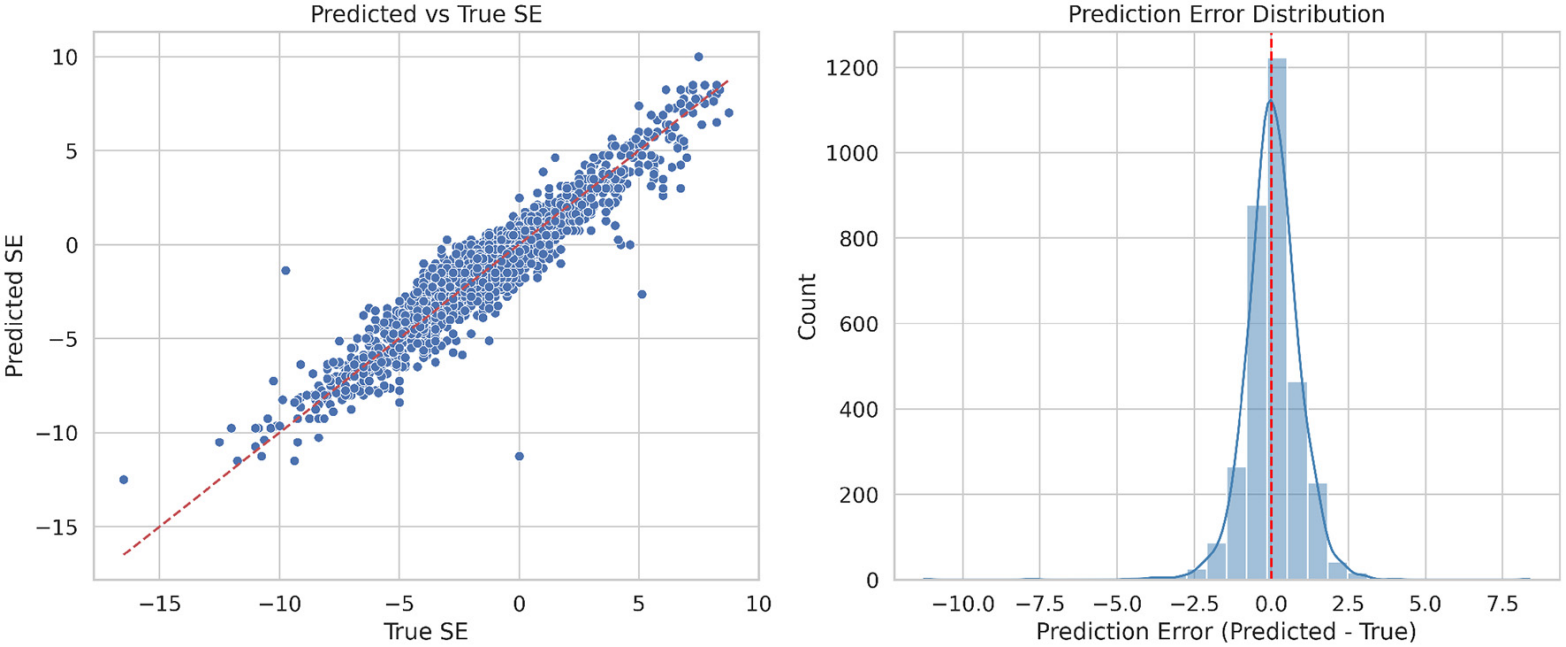
Left: the scatter plot showing predicted versus true spherical equivalent (SE) values in all samples. Right: the error distribution plot in all samples.

To evaluate the model's performance, we analyzed the scatter plot of predicted versus true SE values and the corresponding error distribution for the full test set (Figure 4). Each point represents a sample, and the red dashed line indicates the identity line (Predicted = True). The clustering of points around this line indicates that the model achieves good overall accuracy. The error distribution is approximately Gaussian and centered near zero, suggesting that most predictions have small errors. A few deviations are observed, which may be due to limited image cues or reduced biometric variability in certain refractive ranges. Compared with subgroup analyses, the full dataset exhibits a clearer linear trend and more consistent error distribution, demonstrating improved robustness across the entire spectrum of refractive errors.

**Figure 5:**
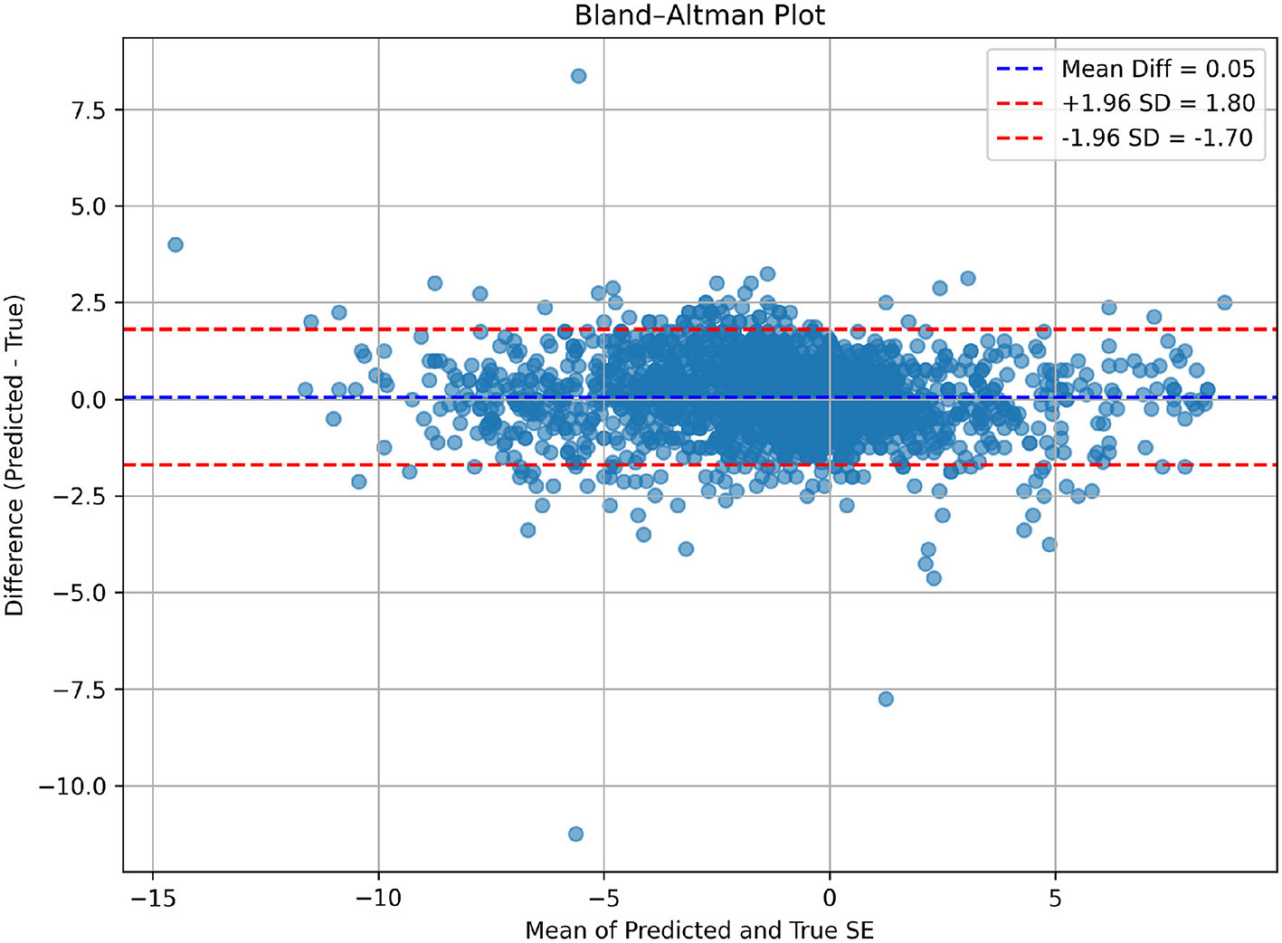
Bland-Altman plot illustrating the agreement between predicted and true SE values in all samples.

Bland-Altman plots for the overall test set (Figure 5) further confirm the agreement between predicted and actual SE values. The mean bias is close to zero (0.05 D), and most points lie within the 95% limits of agreement (-1.7 D to +1.8 D), indicating no systematic over or underestimation and good overall consistency. While some subgroups (e.g., moderate to high myopia or hyperopia) may exhibit slightly wider limits of agreement, the full test set demonstrates tighter agreement and more evenly distributed residuals, further supporting the model's reliability.

### 3.2 Performance stratified by gender

Table 3 shows the prediction performance of the multimodal model across gender subgroups. The model achieved comparable accuracy for both females and males. Specifically, the MAE was 0.6394 D for females and 0.6544 D for males, while RMSE values were 0.8835 D and 0.9063 D, respectively. The coefficient of determination (*R*^2^) remained high for both groups, slightly higher in females (0.8860) than males (0.8807). These results suggest that the model generalizes well across genders, with no significant performance bias observed between male and female subgroups.

**Table 3:** Performance of the multimodal refractive error prediction model stratified by gender.

**Gender**	**Numbers**	**MAE (D)**	**RMSE (D)**	***R* ^2^**	**Pearson r**
Female	1575	0.639	0.884	0.886	0.942
Male	1671	0.654	0.906	0.881	0.939

The evaluation was conducted on the test set, with separate results reported for male and female subgroups. All values are expressed in diopters (D).

### 3.3 Ablation study on input modalities

To evaluate the contribution of each modality, we conducted a systematic ablation study by training separate models from scratch for each modality configuration, rather than masking inputs in a jointly trained network. Specifically, three models were independently trained using identical network architectures, optimization settings, and training protocols. Fundus only model, using retinal fundus images as the sole input; Clinical metadata only model, using non-imaging clinical variables; Multimodal model, jointly incorporating fundus images and clinical parameters. All models were initialized randomly and trained on the same training and validation splits to ensure fair comparison. Performance differences therefore reflect the intrinsic contribution of each modality, rather than information leakage from joint training.

Results are summarized in Table 4. The model using fundus images alone yielded an MAE of 0.9170 D and an *R*^2^ of 0.7602. Clinical metadata alone improved MAE to 0.7604 D and had the *R*^2^ (0.8459), indicating that the metadata contributed complementary information but not sufficient to model the full variance. The multimodal approach, integrating both fundus images and clinical features, achieved the best performance with an MAE of 0.6470 D, RMSE of 0.8951 D, and *R*^2^ of 0.8833. These results clearly demonstrate the benefit of multimodal fusion, with significant improvements in prediction accuracy and model fit when combining structural and clinical information.

**Table 4:** Ablation study comparing model performance across different input modalities: fundus images only, clinical metadata only, and a multimodal combination of both.

**Input modality**	**MAE (D)**	**RMSE (D)**	***R* ^2^**	**Pearson r**
Fundus image only	0.917	1.283	0.760	0.873
Clinical metadata only	0.760	1.029	0.846	0.921
w/o axial length	0.968	1.740	0.559	0.781
Both	0.647	0.895	0.883	0.940

To assess whether the observed performance differences between models were statistically significant, we conducted Wilcoxon signed-rank tests on paired samples. Pairwise comparisons were performed between the multimodal model and each unimodal model. A p-value < 0.05 was considered statistically significant. Wilcoxon signed-rank tests revealed that the performance improvements of the multimodal model were statistically significant when compared with the Fundus image only model (*p* = 6.93 × 10^−56^) and the Clinical metadata only model (*p* = 1.56 × 10^−23^).

To assess the dependence of model performance on axial length (AL), we conducted an additional ablation experiment in which AL was excluded from the input features. A new multimodal model was retrained from scratch using the remaining modalities, including fundus images, age, gender, and corneal curvature. The training protocol, hyperparameters, and evaluation procedures were kept identical to those of the full model to ensure fair comparison. As shown in the ablation results (Table 4), removing axial length led to a moderate decrease in predictive accuracy, indicating that axial length contributes substantially to refractive error estimation. However, the model without axial length maintains reasonable performance, suggesting that the proposed framework can operate under reduced-input settings when axial length is unavailable.

### 3.4 External temporal validation

To further evaluate the generalizability of the proposed model beyond internal validation, an independent external dataset was collected from the same clinical center but during a non-overlapping later time period (July 2025 to October 2025). This temporal split serves as a pseudo-external validation to assess model robustness under real-world data drift conditions. The external validation cohort comprised 252 clinical records collected using the same inclusion criteria and measurement protocols as the primary dataset. Importantly, these samples were not involved in model training, hyperparameter tuning, or internal testing. The trained model was directly applied to this cohort without any further fine-tuning. On the external validation dataset (*n* = 252), the model demonstrated performance comparable to that observed in the internal test set. The error distribution and agreement patterns were consistent across cohorts.

**Figure 6:**
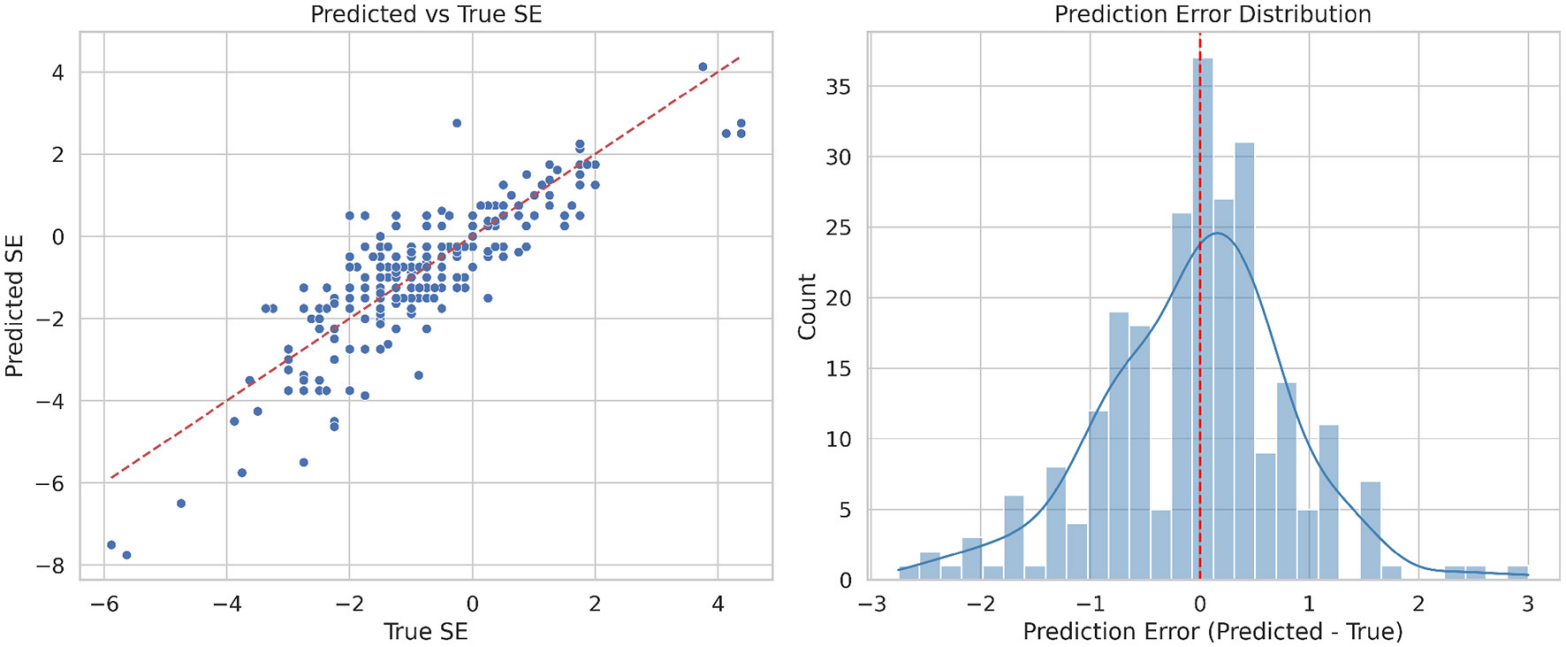
**Left**: the scatter plot showing predicted versus true spherical equivalent (SE) values in the external validation dataset. **Right**: the error distribution plot in the external validation dataset.

**Figure 7:**
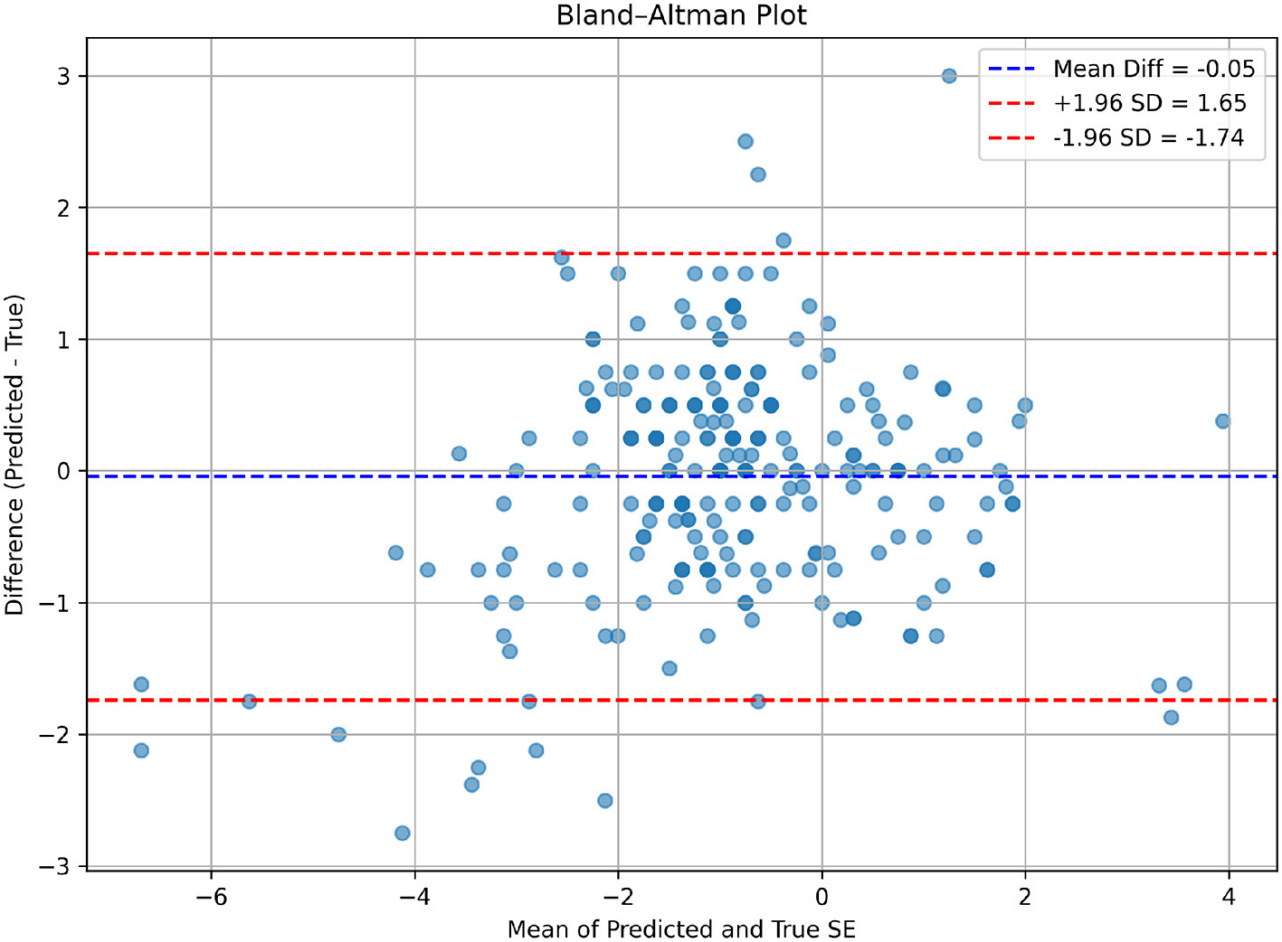
Bland-Altman plot illustrating the agreement between predicted and true SE values in the external validation dataset.

**Figure 8:**
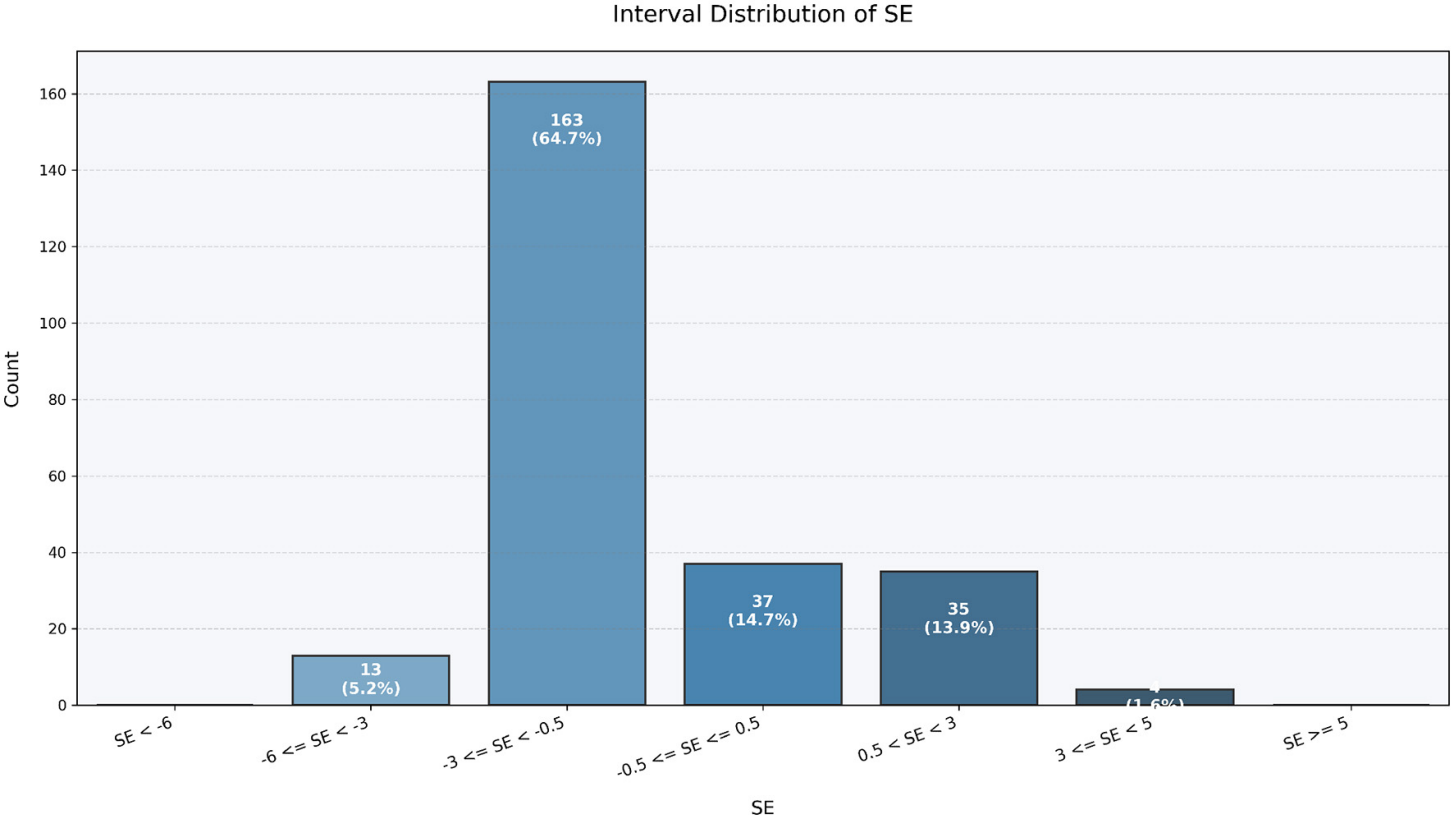
Interval-based distribution of spherical equivalent (SE) values in the external validation dataset.

**Figure 9:**
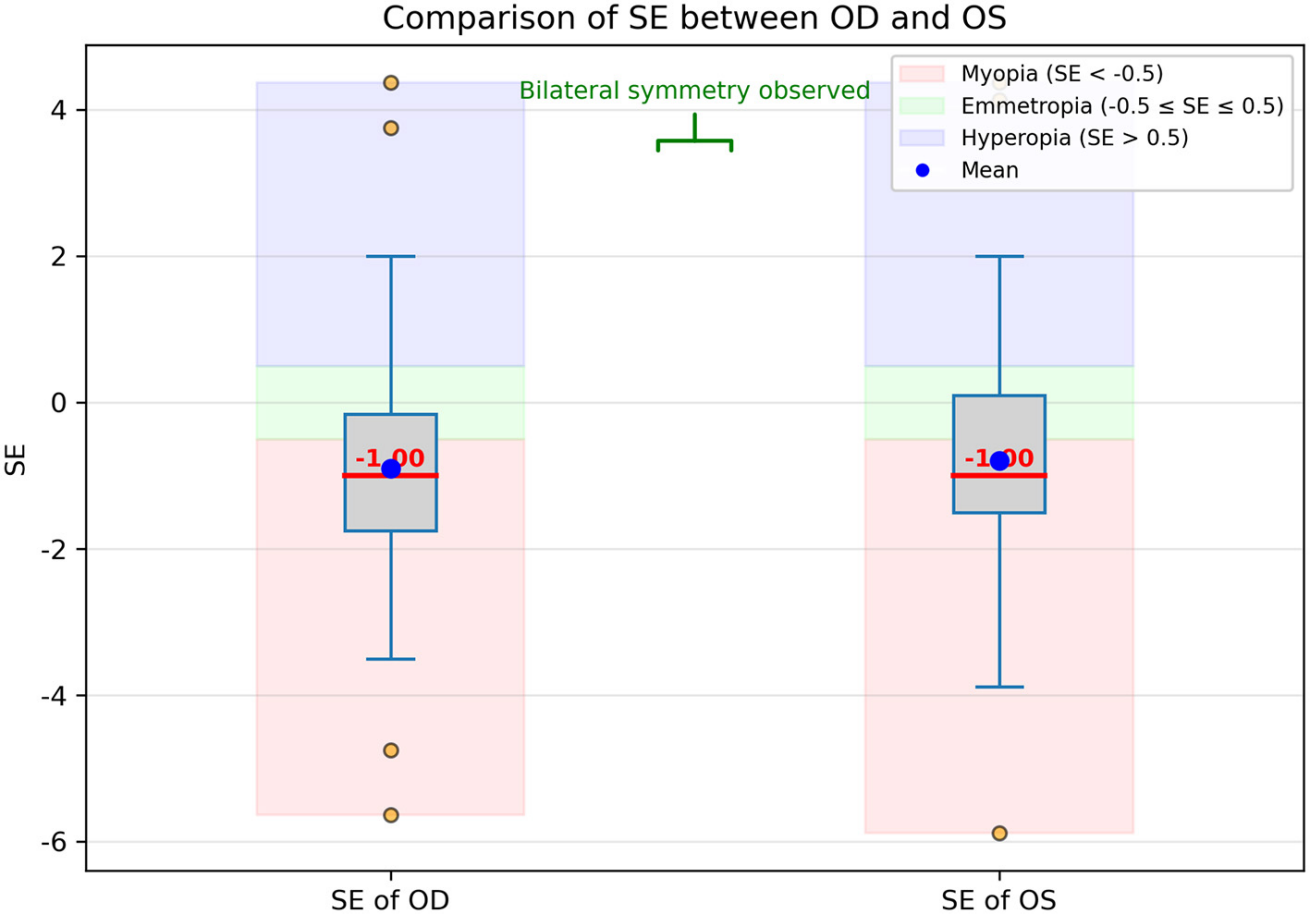
Boxplot comparison of spherical equivalent (SE) values between the right eye (OD) and left eye (OS) in the external validation dataset.

Table 5 shows that the MAE and RMSE values differed only marginally from those obtained in the internal test set. Scatter plots of predicted versus ground-truth spherical equivalent (Figure 6) showed strong concordance, while Bland-Altman analysis (Figure 7) indicated no systematic bias and acceptable limits of agreement. In addition, the refractive error distribution of the external cohort (Figures 8, 9) was similar to that of the internal dataset, suggesting that the model maintained stability under temporal variation in patient data.

**Table 5:** Model performance in the external validation dataset, evaluated using MAE, RMSE, *R*^2^, and Pearson r.

**Numbers**	**MAE (D)**	**RMSE (D)**	***R* ^2^**	**Pearson r**
252	0.653	0.865	0.654	0.847

All error metrics are reported in diopters (D).

## 4 Discussion

This study demonstrates that integrating fundus images with biometric parameters (axial length and corneal curvature) and demographic data can significantly improve the prediction accuracy of spherical equivalent (SE) refractive error in adolescents. The performance gains observed in the multimodal model compared to single-modality models were statistically significant, supporting the value of combining heterogeneous data sources in clinical AI applications.

Although axial length is a strong predictor of refractive error, its measurement requires dedicated biometry devices that may not be universally available in large-scale screening settings. Our ablation results demonstrate that the proposed model can still achieve acceptable performance without axial length, supporting its potential applicability in scenarios where only non-contact imaging and basic demographic information are available. Therefore, while the full model is best suited for clinical or semi-clinical environments, reduced-input variants may enable broader deployment in school-based or community screening programs.

The Bland-Altman analysis revealed minimal systematic bias and narrow limits of agreement across the full dataset, indicating strong concordance with clinically measured SE. However, significantly larger prediction errors and wider limits of agreement were observed in extreme refractive ranges (high myopia and high hyperopia). This finding is consistent with known clinical variability in these groups and may be partly attributable to their underrepresentation in the dataset.

Several limitations should be acknowledged. First, the study population was geographically localized, which may restrict generalizability across diverse ethnic, socioeconomic, and healthcare settings. Second, although the model performed well overall, accuracy decreased in extreme ametropia cases, suggesting that additional data and tailored modeling approaches are needed to better capture rare or complex refractive profiles. Third, the current framework is cross-sectional, limiting its ability to track refractive changes or progression over time. Fourth, the lack of standardized protocols and data formats for AI-based refractive error detection across research projects hinders interoperability and integration into broader health systems. Finally, while spherical error predictions were relatively accurate, cylindrical error (astigmatism) prediction remained less precise, underscoring the need for further refinement.

Although the external validation cohort was obtained from the same clinical center, its temporal separation from the training data provides an important assessment of model robustness under real-world conditions, including potential shifts in patient characteristics and acquisition patterns. Nevertheless, this study remains limited to single-center data, and future work should include multi-center and multi-ethnic cohorts to further confirm generalizability before large-scale deployment.

Future research should prioritize longitudinal studies to enable prediction of refractive progression and support personalized care pathways. Efforts to establish unified standards, shared benchmarks, and interoperable platforms will be critical to facilitate large-scale deployment and equitable access across different healthcare systems. Integrating AI-based vision screening into school health programs and primary care networks could provide scalable, cost-effective solutions for early detection and prevention of myopia and other refractive disorders. Furthermore, enhancing the accuracy of astigmatism prediction and embedding ethical and legal safeguards will strengthen clinical trust and support real-world translation. Collectively, these advances could enable AI-driven refractive error screening to become a cornerstone of digital eye health strategies worldwide.

## 5 Conclusions

In conclusion, statistically significant improvements in SE prediction accuracy were achieved through multimodal integration, but limitations remain in extreme refractive subgroups and generalizability. Future work should focus on expanding population diversity, addressing high ametropia performance, and extending the framework to longitudinal prediction.

## Data Availability

The raw data supporting the conclusions of this article will be made available by the authors, without undue reservation.
